# Frequent down-regulation of ABC transporter genes in prostate cancer

**DOI:** 10.1186/s12885-015-1689-8

**Published:** 2015-10-12

**Authors:** Rita Demidenko, Deividas Razanauskas, Kristina Daniunaite, Juozas Rimantas Lazutka, Feliksas Jankevicius, Sonata Jarmalaite

**Affiliations:** 1 Division of Human Genome Research Centre, Faculty of Nature Sci., Vilnius University, Ciurlionio 21, Vilnius, LT-03101 Lithuania; 2 Faculty of Medicine, Vilnius University, Ciurlionio 21, Vilnius, LT-03101 Lithuania; 3 Urology Centre, Vilnius University, Santariskiu 2, Vilnius, LT-08661 Lithuania

**Keywords:** Prostate cancer, ABC transporters, *ABCB1*

## Abstract

**Background:**

ATP-binding cassette (ABC) transporters are transmembrane proteins responsible for the efflux of a wide variety of substrates, including steroid metabolites, through the cellular membranes. For better characterization of the role of ABC transporters in prostate cancer (PCa) development, the profile of ABC transporter gene expression was analyzed in PCa and noncancerous prostate tissues (NPT).

**Methods:**

TaqMan Low Density Array (TLDA) human ABC transporter plates were used for the gene expression profiling in 10 PCa and 6 NPT specimens. *ABCB1* transcript level was evaluated in a larger set of PCa cases (*N* = 78) and NPT (*N* = 15) by real-time PCR, the same PCa cases were assessed for the gene promoter hypermethylation by methylation-specific PCR.

**Results:**

Expression of eight ABC transporter genes (*ABCA8*, *ABCB1, ABCC6*, *ABCC9*, *ABCC10*, *ABCD2*, *ABCG2,* and *ABCG4*) was significantly down-regulated in PCa as compared to NPT, and only two genes (*ABCC4* and *ABCG1*) were up-regulated. Down-regulation of ABC transporter genes was prevalent in the *TMPRSS2-ERG-*negative cases.

A detailed analysis of *ABCB1* expression confirmed TLDA results: a reduced level of the transcript was identified in PCa in comparison to NPT (*p* = 0.048). Moreover, the *TMPRSS2-ERG*-negative PCa cases showed significantly lower expression of *ABCB1* in comparison to NPT (*p* = 0.003) or the fusion-positive tumors (*p* = 0.002). Promoter methylation of *ABCB1* predominantly occurred in PCa and was rarely detected in NPT (*p* < 0.001).

**Conclusions:**

The study suggests frequent down-regulation of the ABC transporter genes in PCa, especially in the *TMPRSS2-ERG-*negative tumors.

**Electronic supplementary material:**

The online version of this article (doi:10.1186/s12885-015-1689-8) contains supplementary material, which is available to authorized users.

## Background

ATP-binding cassette (ABC) transporters are transmembrane proteins responsible for the transfer of a wide variety of substrates through the extra- and intra-cellular membranes [1]. Cellular metabolites, lipids, sterols, drugs, and other xenobiotics are known as the substrates for ABC transporters. The human genome contains 51 ABC transporter genes (and pseudogenes) arranged in seven subfamilies and named from A to G [2]. In cancer cells, the over-expression of several ABC transporters is related to an increased efflux of chemotherapeutic drugs and the development of multidrug resistance [3]. The strongest connections with multidrug-resistant cancer have been identified for ABCB1 (also known as MDR1 or P-glycoprotein), ABCC1 (also known as MRP1), and ABCG2 (also known as BCRP or MXR) transporters. However, in prostate cancer (PCa), the highly prevalent male malignancy, the studies of ABC transporters are quite limited. Androgens and other steroid hormones are important for the normal development and maintenance of the prostate gland, but they also participate in prostate tumorigenesis. Androgen deprivation therapy through surgical or medical castration is an effective treatment strategy of PCa, but during the disease progression the sensitivity to residual androgens increases resulting in aggressive forms of castration-resistant PCa. Recent studies revealed strong connections between the steroid efflux capacities of ABC transporters and the progression of PCa [4].

ABCB1 is the most studied ABC transporter in PCa. Differently from other tumors, down-regulation rather than over-expression of the *ABCB1* gene has been identified in PCa [5–7]. Aberrant DNA methylation and histone modifications are the main mechanisms responsible for the inactivation of this locus in PCa [7–9]. *ABCB1* expression has been related to the efflux of androgens from PCa cell lines [10] suggesting that reduced levels of ABCB1 might be responsible for intratumoral androgen accumulation and sustained signaling from androgen receptors (ARs) [4]. ABCA1, another important transporter of the ABC family, was recently shown to be related to the development of aggressive PCa through the impaired efflux of cholesterol, an alternative source for androgen synthesis [11]. Several other ABC transporters were shown as the potent regulators of intracellular levels of steroid metabolites, including androgens and antiandrogens [12–14]. Additionally, expression of several ABC transporters was shown to be regulated by androgens or ARs [15, 16]. These data suggest significant involvement of ABC transporters in the pathogenesis of PCa and encourage more detailed analysis of gene expression of the ABC family in relation to clinical characteristics of PCa.

Gene expression profile of human ABC transporters was explored in cancerous and noncancerous prostate tissue by means of TaqMan Low Density Arrays (TLDA). Gene expression and DNA methylation of the *ABCB1* gene were analyzed in a larger set of cases, and the data were correlated with clinical characteristics of PCa. Assessment of the *TMPRSS2-ERG* transcript status enabled identification of novel associations between this fusion transcript and the expression of ABC transporter genes.

## Methods

### Sample collection and clinical data

Prostate tissue samples were obtained from 104 PSA-screened and biopsy-proven PCa patients treated with a radical prostatectomy (RP) at the Vilnius University Urology Centre from 2008 to 2014. The research was a part of large-scale PCa biomarker study conducted according to standardised protocols of sample collection and processing reported previously [17]. Cancerous (≥70 % of tumor cells) and noncancerous (0 %) prostatectomy tissues were sampled by expert pathologist as previously reported [17] and prepared for molecular analysis. The results of clinical, postoperative pathological and molecular examinations are presented in Table 1. None of these patients had received preoperative radiotherapy, chemotherapy, or hormonal treatment. Approval from the Lithuanian Bioethics Committee was obtained before initiating the study and all patients gave informed consent for participation.

**Table 1:** Characteristics of study group. ABCB1 analysis group was involved in the *ABCB1* gene expression and methylation analysis, while TLDA analysis group was profiled for the ABC transporter genes expression. NPT – noncancerous prostate tissue; PCa – prostate cancer; BCR – biochemical recurrence; PSA – prostate-specific antigen

Variable	ABCB1 analysis group		TLDA analysis group	
	*N* = 93		*N* = 16	
	NPT	PCa	NPT	PCa
	*N* = 15 + 10^a^	*N* = 78	*N* = 6	*N* = 10
Mean age in years ± SEM	62.13 ± 1.01	60.76 ± 0.85	61.33 ± 1.09	62.9 ± 2.04
Pathological stage				
pT2		52		5
pT3		25		5
Gleason score				
6		18		3
≥ 7		54		7
Unknown		1		
BCR				
Yes		20		5
No		53		5
Unknown		5		
*TMPRSS2-ERG* status				
Positive		51		6
Negative		27		4
Mean PSA level at diagnosis, ng/ml ± SEM		10.57 ± 1.25		11.07 ± 3.03
Methylation status of *ABCB1* promoter				
Methylated	4	56		5
Unmethylated	15	22		5
Unknown	6		6	
Mean expression of *ABCB1*, ∆Cq ± SEM	7.50 ± 0.24	8.14 ± 0.13	5.01 ± 0.18	5.61 ± 0.17

^a^Ten additional samples were included in *ABCB1* methylation analysis

Prostate tumors of a Gleason score 6–8 and of an intermediate stage (pT2-pT3) were included in our study (Table 1). Ten PCa and 6 noncancerous prostate tissues (NPT) were screened on human ABC transporter TLDA cards. The *ABCB1* gene expression analysis was performed on 78 PCa tissues and 15 NPT specimens from PCa patients. The same (*N* = 78) PCa specimens and a set of NPT samples (*N* = 9) were analyzed for the *ABCB1* gene promoter DNA methylation. Ten additional NPT samples were included in this analysis, resulting in a control group of 19 NPT specimens. Follow-up data were available for 93.59 % (73/78) of patients with a mean follow-up time of 3 years. Biochemical recurrence (BCR) was defined as a detection of serum PSA level of >0.20 ng/mL by two subsequent measurements after RP. The status of the fusion transcript *TMPRSS2-ERG* was identified as reported previously [17].

### Gene expression analysis with TLDA

Total RNA from snap-frozen sections was isolated with mirVana Kit (Ambion, Life Technologies, Thermo Fisher Scientific Foster City, CA, USA) according to the manufacturer’s recommendations. The quantity of the RNA samples was measured spectrophotometrically using the NanoDrop 2000 (Thermo Fisher Scientific, Wilmington, NC, USA). Integrity (RIN) of the RNA samples was checked with the 2100 Bioanalyzer system (Agilent Technologies, Santa Clara, CA, USA).

Reverse transcription (RT) was done using 500 ng of total RNA and High Capacity cDNA Reverse Transcription Kit with RNase Inhibitor according to the manufacturer’s instructions (Applied Biosystems, Life Technologies, Thermo Fisher Scientific, Foster city, CA, USA).

Gene expression of human ABC transporters was profiled using TLDA cards with the human ABC transporter panel (Applied Biosystems), containing 50 human ABC transporter genes and 14 proposed reference genes. Twenty μL of cDNA were used as a template for the measurement of mRNA in quantitative PCR (qPCR). RT-qPCR was performed using TaqMan Universal Master Mix II, no UNG from Applied Biosystems on the ViiA 7 Real-Time PCR System as recommended by the manufacturer (Applied Biosystems). Thermal cycling conditions were as follows: 95 °C for 10 min, then 95 °C for 15 s and 60 °C for 1 min for 40 cycles.

Raw Cq-values with automatically selected thresholds were calculated using the ViiA 7 version 1.1 software (Applied Biosystems). Expression level of each gene for all samples was analyzed in triplicate and required at least two valid wells. Only genes having ≥70 % of valid data of all samples were involved in further analyses. According to the NormFinder and GeNorm algorithms the combination of *POLR2A*, *PGK1*, *PPIA*, *ACTB*, *B2M*, and *HMBS* was shown as the most suitable set of reference genes and was used for further TLDA data analysis.

### Target gene RT-qPCR

Total RNA from snap-frozen sections was isolated using the phenol-chloroform method. Quantity of the RNA samples was measured spectrophotometrically using the NanoDrop 2000 (Thermo Fisher Scientific). For RT-qPCR, 1 μg of total RNA was converted to cDNA using Maxima First Strand cDNA Synthesis Kit for RT-qPCR (Thermo Fisher Scientific) in a final volume of 20 μL. RT products were amplified on Mastercycler-pro thermocycler (Eppendorf, Hamburg, Germany) under the following conditions: 25 °C for 10 min, followed by 50 °C for 15 min, and the termination of reaction by heating at 85 °C for 5 min. A negative control without reverse transcriptase was included for each sample.

*ABCB1* expression was analyzed by RT-qPCR with SYBR Green labeling using the following primers: F-5´-CCCATCATTGCAATAGCAGG-3´ and R-5´-GTTCAAACTTCTGCTCCTGA-3´. *GAPDH* was used as a reference gene and the product was amplified with the primers: F-5´-GAAGGTCGGAGTCAACGGATTT-3´and R-5´-ATGGGTGGAATCATATTGGAAC-3´ (all from Metabion International AG, Martinsried, Germany).

RT-qPCR mix was prepared using Maxima SYBR Green qPCR Master Mix (2X) (Thermo Fisher Scientific), 0.3 μM forward and reverse primers, and 2 μL cDNA in a total volume of 25 μL. Well-to-well variation was normalized by adding 10 nM ROX. QPCR was performed on the Viia7 Real-Time PCR System (Applied Biosystems) under the following conditions: enzyme activation at 50 °C for 2 min followed by 95 °C for 10 min, then amplification at 95 °C for 15 s and 60 °C for 1 min for 40 cycles in total, and the melting step at default parameters. Cq values were calculated with Viia7 software version 1.1 (Applied Biosystems). All gene assays were measured in duplicate and required at least two valid wells. A negative control without cDNA was included for each primer pair in every RT-qPCR run.

### DNA methylation analysis

Up to 20 mg of prostate tissue were digested with proteinase-K and DNA was extracted using standard phenol-chloroform protocol followed by ethanol precipitation. Modification with sodium bisulfite was performed using EZ DNA Methylation Kit (Zymo Research, Irvine, CA, USA) according to the manufacturer’s protocol.

Bisulfite-modified DNA was used as a template for methylation-specific PCR (MSP) with *ABCB1* primers [18] specific for either methylated or unmethylated DNA. The MSP reaction mix (25 μL) contained 1x AmpliTaq Gold Buffer, 2.5 mM MgCl_2_, 1.6 mM dNTP mix, 1.25 U AmpliTaq Gold 360 DNA Polymerase (Applied Biosystems), 0.5 μM of each primer, and 1 μL of bisulfite-modified DNA. Amplification conditions were as follows: 95 °C for 10 min, then 37 cycles at 95 °C for 45 s, 60 °C for 45 s, and 72 °C for 45 s, and final extension at 72 °C for 10 min. The MSP products were run on 3 % agarose gel with ethidium bromide staining. Bisulfite-modified leukocyte DNA from healthy donors served as a negative control for methylated DNA and SssI methylase-treated (Thermo Fisher Scientific) bisulfite-modified leukocyte DNA served as a positive control. Non-template controls were included in each PCR run.

### Statistical analysis

Computation of statistical tests was performed using GraphPad Prism version 5.00 for Windows (GraphPad Software, San Diego California USA) and GenEx version 6.0.1 software (MultiD Analyses AB, Göteborg, Sweden). Survival analysis of the TLDA cohort was carried out with MedCalc Statistical Software version 14.12.0 (MedCalc Software, Ostend, Belgium). A t-test indicated relative gene expression differences between two groups. For methylation data, a Mann–Whitney U-test (for continuous variables) and a two-tailed Fisher’s exact test (for categorical variables) were applied to identify statistically significant differences between groups. Spearman’s correlation coefficient was calculated to identify significant associations. P-values of less than 0.05 were considered significant.

## Results

### Gene expression profile of ABC transporters in PCa

In order to explore the expression levels of the ABC genes, 10 PCa and 6 NPT samples were profiled on human ABC transporter TLDAs containing probes for 50 ABC genes in total. Expression of 45 genes was consistently identified in prostate tissue, with the highest levels (mean Cq < 24) characteristic to *ABCA11*, *ABCC4*, *ABCD3*, *ABCE1*, and *ABCF3*. In contrast, the *ABCB5*, *ABCC12, ABCC13*, *ABCG5,* and *ABCG8* genes showed low or undetectable expression (mean Cq > 35) in prostate tissue and were eliminated from further analysis.

**Fig. 1:**
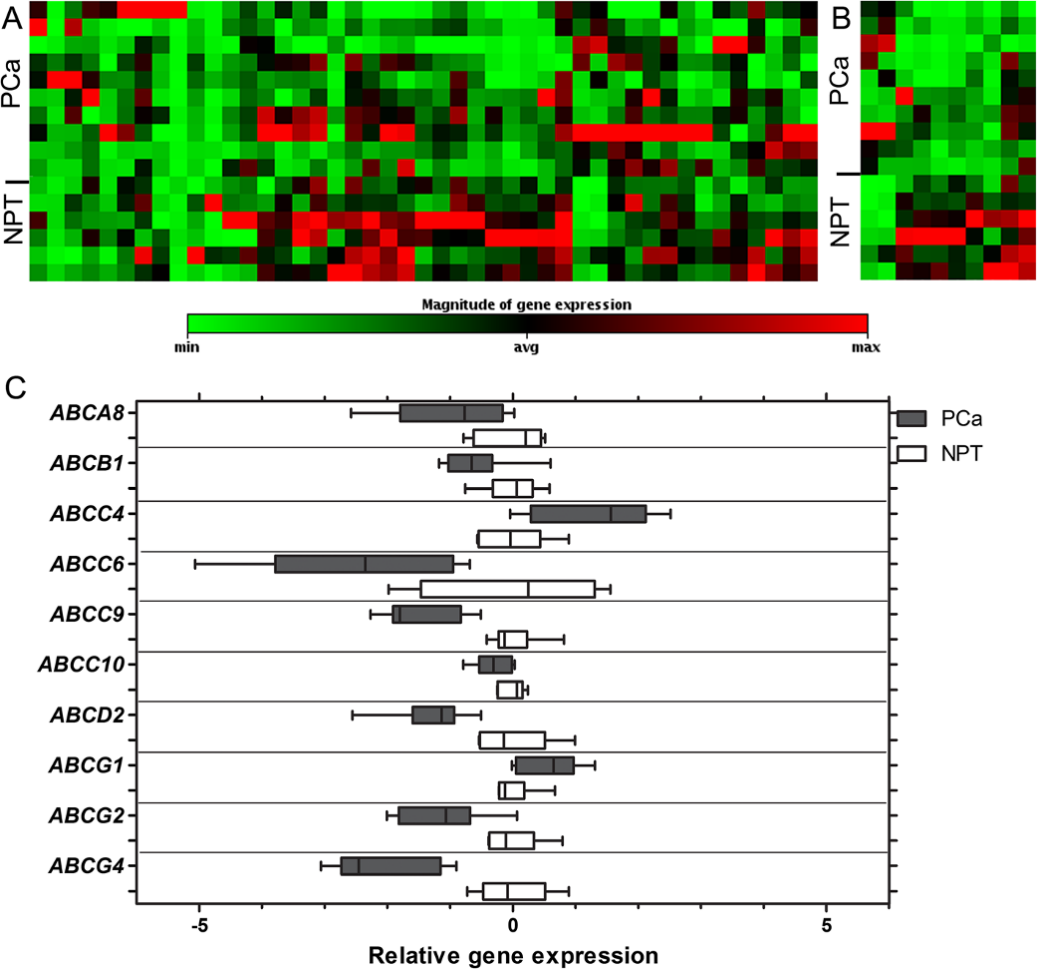
Profile of ABC transporter gene expression in prostate cancer (PCa) and noncancerous prostate tissue (NPT). Heat maps represent expression profile of all analyzed (**a**) and significantly (*p* < 0.05) deregulated (**b**) ABC genes. Box plots (**c**) show expression levels of significantly deregulated ABC genes. The box extends from the 25^th^ to 75^th^ percentiles. The line in the middle of the box is plotted at the median. The whiskers indicate the smallest and largest values

Comparison of PCa to NPT revealed a specific ABC gene expression signature of PCa characterized by marked down-regulation of several ABC transporter genes (Fig. 1a). Eight ABC transporter genes were significantly down-regulated in cancerous prostate tissues (Fig. 1b and c), including *ABCA8* (FC 2.03; *p* = 0.029), *ABCB1* (FC 1.52; *p* = 0.035), *ABCC6* (FC 5.67; *p* = 0.005), *ABCC9* (FC 2.77; *p* < 0.001), *ABCC10* (FC 1.24; *p* = 0.030), *ABCD2* (FC 2.44; *p* < 0.001), *ABCG2* (FC 2.22; *p* = 0.003), and *ABCG4* (FC 2.14; *p* < 0.001; Fig. 1c). Only two out of 45 analyzed ABC transporter genes, namely *ABCC4* (FC 2.49; *p* = 0.007) and *ABCG1* (FC 1.48; *p* = 0.029), were up-regulated in PCa in comparison to NPT (Fig. 1b and c, Additional file 1).

**Fig. 2:**
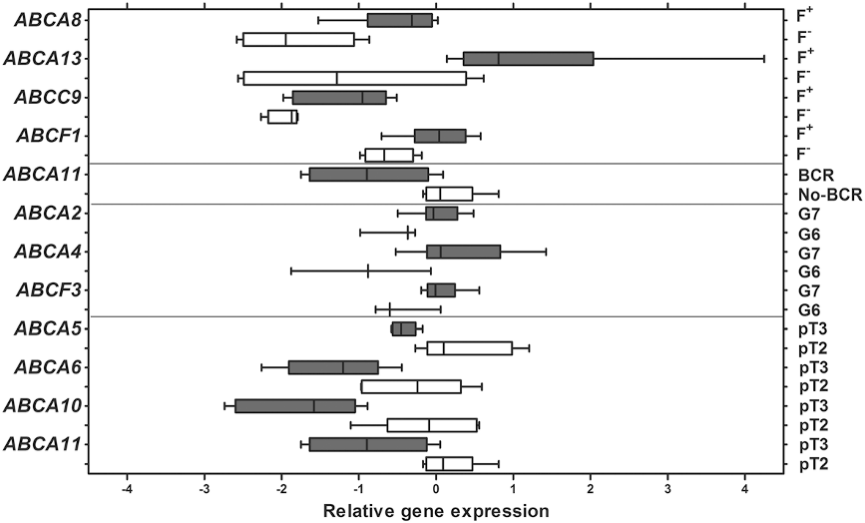
Significant changes (*p* < 0.05) of ABC transporter gene expression in PCa according to selected variables. F^+/−^ − *TMPRRS2-ERG* fusion status; BCR – biochemical recurrence; G7/6 – Gleason score 7/6; pT3/2 – pathological tumor stage 3/2. The box extends from the 25^th^ to the 75^th^ percentiles. The line in the middle of the box is plotted at the median. The whiskers indicate the smallest and largest values

Remarkably, a quite different profile of ABC gene expression was observed in PCa stratified according to the *TMPRSS2-ERG* transcript status. *ABCA8* and *ABCC9*, the genes that were significantly down-regulated in PCa in comparison to NPT, showed high expression in the *TMPRSS2-ERG-*positive cases, but were suppressed in the fusion-negative cases. The total list of ABC transporters that were down-regulated in the *TMPRSS2-ERG-*negative cases included *ABCA8* (FC 2.56; *p* = 0.012), *ABCA13* (FC 5.35; *p* = 0.039), *ABCC9* (FC 1.74; *p* = 0.037), and *ABCF1* (FC 1.57; *p* = 0.038; Fig. 2, Additional file 1).

PCa cases that experienced BCR (*N* = 5) showed significant reduction of the *ABCA11* gene expression (FC 2.03; *p* = 0.031; Additional file 1). The expression level of *ABCA11* was negatively correlated with tumor size (R −0.83; *p* = 0.003) and extra-prostatic tumor stage (FC 2.05; *p* = 0.028). Univariate survival analysis revealed borderline significance of *ABCA11* expression (*p* = 0.072) in predicting BCR.

In addition to *ABCA11*, the extra-prostatic tumor stage (pT3 vs pT2) was associated with the down-regulation of other ABC transporter genes from subfamily A (Fig. 2, Additional file 1): *ABCA5* (FC 1.73; *p* = 0.022), *ABCA6* (FC 2.00; *p* = 0.046), and *ABCA10* (FC 3.29; *p* = 0.006). In contrast, the higher Gleason score (Gleason 7 vs 6) was related to over-expression of several genes: *ABCA2* (FC 1.47; *p* = 0.041), *ABCA4* (FC 2.41; *p* = 0.038), and *ABCF3* (FC 1.44; *p* = 0.041). This might be explained by the predominance of the *TMPRSS2-ERG*-positive cases among tumors with Gleason score 7.

### *ABCB1* expression and DNA methylation in PCa

**Fig. 3:**
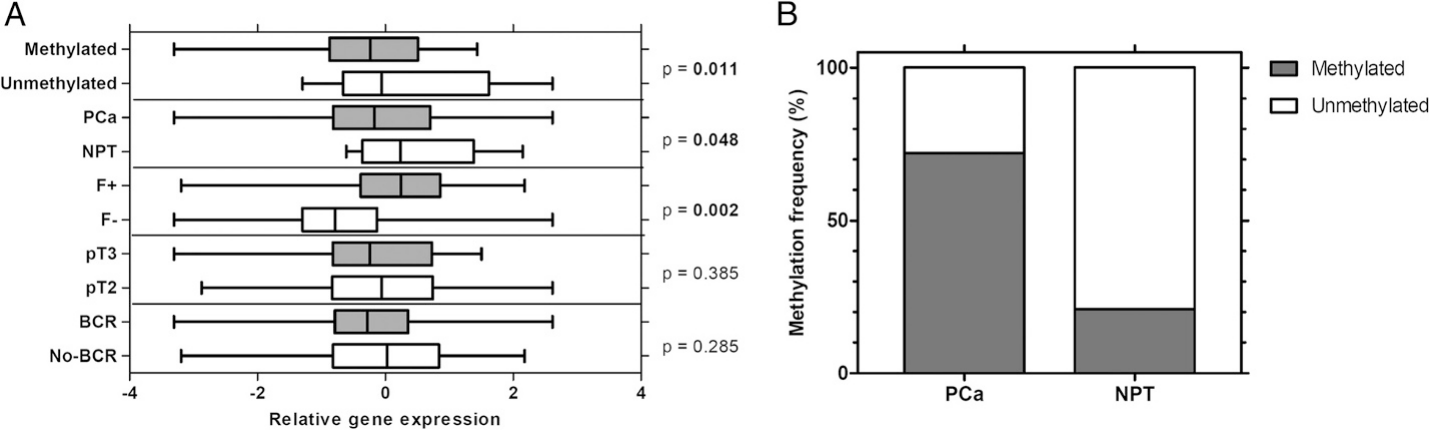
Expression levels (**a**) and methylation frequency (**b**) of *ABCB1* in prostate tissues. PCa – prostate cancer; NPT – noncancerous prostate tissue; BCR – biochemical recurrence; F^+/−^ – *TMPRRS2-ERG* fusion status. The box of figure A extends from the 25^th^ to the 75^th^ percentiles. The line in the middle of the box is plotted at the median. The whiskers indicate the smallest and largest values

For the verification of TLDA results, expression of the *ABCB1* gene was evaluated in a larger group of PCa cases (*N* = 78) and NPT (*N* = 15) specimens. As in the TLDA analysis, the *ABCB1* expression was significantly lower in PCa than in NPT tissues (FC 1.56; *p* = 0.048; Fig. 3a). Moreover, the *TMPRSS2-ERG*-negative PCa cases showed significantly lower *ABCB1* expression in comparison to the fusion-positive tumors (FC 1.77; *p* = 0.002) or NPT (FC 2.90; *p* = 0.003). A negative association (R −0.29; *p* = 0.012) was detected between *ABCB1* expression and the preoperative PSA level. No other significant correlations with clinical variables (pT or Gleason score) were identified, and similar levels of *ABCB1* were observed in BCR and no-BCR cases (Fig. 3a).

The MSP analysis (Fig. 3b) was applied for the detection of aberrant DNA methylation in the promoter region of the *ABCB1* gene in the same set of PCa cases. More than 70 % of PCa tissues (56/78; 71.79 %) showed aberrant *ABCB1* promoter methylation, while this change rarely occurred in NPT specimens (4/19; *p* < 0.001). Comparison to gene expression data revealed statistically significantly lower levels the *ABCB1* transcript in the PCa tissues with the promoter hypermethylation as compared to the cases without hypermethylation (FC 1.66; *p* = 0.011; Fig. 3a)*.* Similarly to the gene expression, no statistically significant correlations between the *ABCB1* methylation status and clinical variables were identified.

### CpG islands of the ABC transporter genes

To test the possible involvement of DNA methylation in transcriptional regulation of other ABC genes, CpG islands (CGI) of the significantly deregulated ABC promoters were characterized based on data provided by the NCBI Epigenomics browser. For genes with no CGIs present, the DBCAT online tool [19] was used to predict potential CGIs, applying the following parameters: observed/expected CpG ratio 0.6, minimal CGI length 300 bp, and GC content ≥50 %. Promoter regions were obtained from the Swiss Regulon Portal [20] or from the Eukaryotic Promoter Database [21]. Typical CGIs were identified in 5’ regions in 12 out of 19 (74 %) significantly deregulated ABC genes, and 2 additional CGIs were predicted using CGI detection tool (Table 2). Mean length of the identified CGIs was more than 1 kb, the GC content exceeded 70 %, and most of the CGIs were located in the promoter regions of the ABC genes. Only two out of these 14 CGIs of the ABC genes (*ABCB1* and *ABCG2*) have been studied for DNA methylation changes in PCa [7–9, 22–24], while others deserve further investigations.

**Table 2:** Characterization of CpG islands (CGIs) of significantly deregulated ABC transporter genes. CGI locations are provided relative to transcription start site (TSS). Gene expression changes were identified in comparisons: Gleason score 7 versus 6 (G7/6); tumor stage 3 versus 2 (pT3/2); prostate cancer versus noncancerous prostate tissue (PCa/NPT); *TMPRSS2-ERG* fusion transcript negative versus positive cases (F^−/+^)

Gene name	Gene expression	Group comparison	Gene location	CGI presence	GC content, %	CGI length, bp	CGI location according to TSS
*ABCA2*	Up	G7/6	9q34	Yes	78	1196	+134/+1329
*ABCA4*	Up	G7/6	1p22	No	–	–	–
*ABCA5*	Down	pT3/2	17q24.3	Yes	67	1319	−669/+650
*ABCA6*	Down	pT3/2	17q24.3	No	−	−	−
*ABCA8*	Down	PCa/NPT; F^−/+^	17q24	No	−	−	−
*ABCA10*	Down	pT3/2	17q24	No	−	−	−
*ABCA11*	Down	BCR+/−; pT3/2	4p16.3	Yes	63	724	−10/+714
*ABCA13*	Down	pT3/2; F^−/+^	7p12.3	Yes^a^	62	618	+283291/+283908
*ABCB1*	Down	PCa/NPT; F^−/+;^ pT3/2	7q21.12	Yes	57	1048	+112174/+113221
*ABCC4*	Up	PCa/NPT	13q32	Yes	68	1355	−849/+506
*ABCC6*	Down	PCa/NPT	16p13.1	Yes^a^	58	1397	−1022/+375
*ABCC9*	Down	PCa/NPT; F^−/+^	12p12.1	Yes	56	1019	−733/+286
*ABCC10*	Down	PCa/NPT	6p21.1	Yes	59	964	−454/+510
*ABCD2*	Down	PCa/NPT	12q12	No	−	−	−
*ABCF1*	Down	F^−/+^	6p21.33	Yes	58	1222	−655/+567
*ABCF3*	Up	G7/6	3q27.1	Yes	62	737	−269/+468
*ABCG1*	Up	PCa/NPT	21q22.3	Yes	67	1709	−437/+1272
*ABCG2*	Down	PCa/NPT	4q22	Yes	65	1053	−589/+464
*ABCG4*	Down	PCa/NPT	11q23.3	Yes	73	888	−262/+626

^a^Predicted CGI

## Discussion

Current evidence suggests a role for membrane transporters in control of intratumoral androgen level important for PCa development and progression [10–14]. In the present study, for the better characterization of the role of ABC transporters in prostate tumorigenesis, expression levels of all human ABC transporter genes were evaluated for the first time in cancerous and noncancerous prostate tissue. This study identified a specific profile of ABC gene expression in PCa characterized by the down-regulation of several ABC genes. Deregulated expression of a set of ABC genes was particularly evident in the *TMPRSS2-ERG*-negative prostate tumors.

Expression of 45 out of 50 ABC transporter genes loaded on the array was identified in prostate tissue, while five genes showed low or undetectable levels of expression. Transcription levels of eight ABC transporter genes, including *ABCA8*, *ABCB1*, *ABCC6*, *ABCC9*, *ABCC10*, *ABCD2*, *ABCG2*, and *ABCG4,* were significantly down-regulated in PCa, and only two genes, *ABCC4* and *ABCG1*, were up-regulated. In a larger set of PCa cases, the expression of *ABCB1* was also significantly reduced in PCa relative to NPT, and this suppression was associated with increased preoperative PSA level. Our data are in agreement with other studies of ABC transporters in PCa. Several studies reported the down-regulation of the *ABCB1* gene [5–7] and reduced levels of protein expression [9] in PCa in comparison to NPT. Similarly, loss of the ABCA1 protein expression was detected in PCa, especially in the higher grade tumors [11]. The ABCA5 protein was detectable in basal cells of normal prostate glands and premalignant lesions, but was faintly expressed in prostate cancer glands [25]. Down-regulation of the ABCC4 or ABCG2 transporters was recently reported in PCa [16, 23]. Expression of *ABCC4* was shown to be reduced after androgen ablation, and castration-resistant PCa cases had lower levels of this transporter [15, 16]. Expression or functional activities of the remaining members of this large gene family are mainly unexplored in PCa.

DNA hypermethylation is a powerful mechanism of gene inactivation. In PCa, aberrant DNA methylation of the *ABCB1* promoter was reported in several studies, and correlations with reduced gene or protein expression were identified [7–9, 26, 27]. In agreement with these previous studies, our data showed frequent (72 %) hypermethylation of the *ABCB1* promoter in PCa, and significant association between the aberrant methylation and reduced expression of the transcript. Similarly, the loss of expression of *ABCA1*, another transporter of the ABC family, was recently [11] related to the aberrant methylation of the promoter region in PCa. Besides, comparison of the global methylation pattern of genes in PCa cell lines and normal prostate cell lines [7] revealed predominant hypermethylation of different membrane transporter genes in PCa cell lines. Among them, two genes of the ABC family, *ABCB1* and *ABCC7*, showed increased methylation in PCa cell lines. Similarly, hypermethylation of the *ABCC6* gene was identified in urine of bladder cancer patients [28]. This suggests that frequent down-regulation of ABC transporter genes in PCa, observed in our study and in other publications [5–7, 11, 16, 23] might be caused by the gain of DNA methylation in ABC gene loci. In support of this concept, strong CpG islands were identified in loci encoding 14 out of 19 ABC genes that were deregulated in PCa tissues in our study. This observation encourages further investigation of epigenetic aberrations of ABC transporter genes in PCa as a possible mechanism of prostate tumorigenesis and PCa progression.

Although the *TMPRSS2-ERG* fusion is the most prevalent genetic rearrangement found in approximately 50 % of PCa [29], the clinical implications of this gene fusion are still unclear. The fusion transcript is usually composed of the androgen-sensitive *TMPRSS2* promoter and the *ERG* sequence. This genetic rearrangement results in the androgen-regulated oncogenic transcription factor. In our study, despite the predominant down-regulation of ABC transporters in PCa specimens, the *TMPRSS2-ERG* fusion-positive tumors showed quite high levels of *ABCB1* and several other ABC genes in comparison to the fusion-negative cases. cMYC, which is a direct transcriptional regulator of a large set of ABC transporters, is usually over-expressed in the fusion-positive PCa [30] and might be responsible for this *TMPRSS2-ERG* fusion-related ABC gene expression profile. More importantly, our study revealed marked down-regulation of four ABC transporter genes (*ABCA8*, *ABCA13*, *ABCC9*, and *ABCF1)* in the subgroup of PCa cases that were negative for the *TMPRSS2-ERG* transcript. In addition, the expression of *ABCB1* was also significantly reduced in the *TMPRSS2-ERG*-negative cases in our validation study. The altered expression of ABC transporters has been shown to reduce the efflux of androgens and their precursors [4], while intracellular accumulation of androgens is responsible for sustained AR signaling. This ABC transporters deficiency-related AR activation might serve as an important pathway of tumorigenesis in *TMPRSS2-ERG*-negative cases. Moreover, these changes in ABC gene expression might favor development of a progressive, anti-androgen therapy-resistant phenotype of PCa. However, the exact mechanism and consequences of the down-regulation of ABC transporter genes in PCa need to be clarified in functional studies.

PCa is a highly variable disease with multiple genetic and epigenetic alterations affecting a wide range of biological pathways. During recent years multiple molecular markers of PCa have been explored, however, the implication of ABC transporters in prostate cancerogenesis is still poorly understood. Besides extensively documented hypermethylation of the *ABCB1* promoter, our study demonstrates that down-regulation of other ABC transporter genes occurs in PCa. Deregulated expression of several ABC genes shows significant associations with advanced tumor stage, grade, other clinical variables, and *TMPRSS2-ERG* status. Moreover, our data indicate that a set of these significantly deregulated ABC genes possess strong CGIs in their promoters and might be controlled by DNA methylation and other epigenetic phenomena. Significantly deregulated ABC genes identified by our TLDA-based screening warrant further investigation for their diagnostic and prognostic potential in PCa, whereas epigenetic therapy might be considered for treatment of androgen deprivation therapy-resistant tumors.

## Conclusions

In prostate tumors, expression of several ABC transporter genes is down-regulated and shows significant associations with clinical variables and the absence of the *TMPRSS2-ERG* fusion transcript. *ABCB1* analysis and characterization of CpG islands of the ABC loci suggest aberrant DNA methylation as a plausible mechanism inactivating expression of the ABC transporter genes in PCa.

## Additional file

Additional file 1: **Significant changes (*****p***** < 0.05) of ABC transporter gene expression in different comparisons of subgroups.** PCa – prostate cancer; NPT – noncancerous prostate tissue; BCR – biochemical recurrence; *TMPRSS2-ERG*+/- – fusion transcript positive/negative cases; pT – tumour stage; FC – fold change. (XLSX 10 kb)
